# Gas-to-Particle Conversion in Surface Discharge Nonthermal Plasmas and Its Implications for Atmospheric Chemistry

**DOI:** 10.3390/s110302992

**Published:** 2011-03-07

**Authors:** Hyun-Ha Kim, Atsushi Ogata

**Affiliations:** National Institute of Advanced Industrial Science and Technology (AIST), AIST Tsukuba West, 16-1 Onogawa, Tsukuba, Ibaraki 305-8569, Japan; E-Mail: atsushi-ogata@aist.go.jp

**Keywords:** nonthermal plasma, aerosol formation, secondary organic aerosol (SOA), volatile organic compound (VOC)

## Abstract

This paper presents some experimental data on gas-to-particle conversion of benzene using nonthermal plasma (NTP) technology and discusses the possibility of its technical application in atmospheric chemistry. Aerosol measurement using a differential mobility analyzer (DMA) revealed that the parts of benzene molecules were converted into a nanometer-sized aerosol. Aerosol formation was found to be highly related with the missing part in carbon balance. Scanning electron microscopy analysis showed that the aerosols formed in synthetic humid air are the collection of nanoparticles. The carbonyl band (C=O) was found to be an important chemical constituent in the aerosol. The potential of the NTP as an accelerated test tool in studying secondary organic aerosol (SOA) formation from VOCs will be also addressed.

## 1. Introduction

The emission of volatile organic compounds (VOCs) into open air is of great importance in terms of photochemical smog and secondary organic aerosols (SOA). These two air pollution events occur at the same time, mostly in urban areas. Aerosol formation in the troposphere in particular leads to degraded visibility and has direct health effects on human beings. VOC emission regulations in many countries are aimed basically at the reduction of these two problems. VOC-related chemistry and its potential for SOA formation have been the subject of intensive studies in the past three decades [1].

A nonthermal plasma (NTP) is a partially ionized gas which can induce various chemical reactions, even at room temperature and atmospheric pressure. In contrast to the thermal plasma where all the components are at thermal equilibrium (usually around 10,000 K), an NTP is characterized by the different energy states between electrons, ions and neutral molecules. Because of their small mass electrons can be easily accelerated under the influence of electric fields and attain kinetic energies of up to 20 eV. These energetic electrons ionize and dissociate background molecules, resulting in the formation of highly reactive chemical species (radicals, ions, excited molecules and ozone). Ozone generation is one good example of a nonthermal plasma chemical reaction, which is used extensively in various industries [2,3]. NTP has also been considered as a control technology for various air pollutants such as SOx, NOx and VOCs [4]. One of important issues in plasma technology is the formation of unwanted byproducts including aerosols. Most recent work on VOC removal look at the combination of NTP with a catalyst due mostly to the concerns about energy efficiency and byproducts [5,6]. The plasma chemical reactions are based on the gas-phase radical reactions involving chemically active species (CAS) such as atomic oxygen, hydroxyl radicals, peroxy radicals and ozone, which is quite similar to atmospheric chemistry. The extensive database on chemical reactions involving CAS has also been used in modeling plasma chemical reactions. Table 1 compares the major chemical components in the NTP process and the atmospheric chemistry. Although a chamber test provides reliable data on the photochemical reactions under controlled reaction conditions, which are similar to those in photochemical smog episodes, the major drawbacks are the large facility and long reaction time [7,8]. On the other hand, gas-to-particle conversion in an NTP takes place on a short time scale due to the high concentrations of chemically reactive species. The concentrations of these species are 3–8 orders of magnitude larger than those observed in atmospheric chemistry. It is also of interesting, from the viewpoint of practical applications of NTP as a tool for atmospheric chemistry, to study the formation of SOA, *i.e.*,

1. one can easily control the reaction rate by adjusting the energy input to the reactor,
2. it requires only a simple and compact reaction chamber, and
3. it can be easily prepared and coupled with various on-line measurement instruments.

Gas-to-particle conversion in plasma chemical reactions has been observed by the formation solid products on the surface, which is referred to as polymerization [9,10]. In recent years, Anderson *et al.* observed polymer deposition in the decomposition of 5,370 ppm styrene using a silent discharge plasma reactor in an Ar/O_2_ mixture [11]. Polymerization of phenol vapor was studied in a dielectric-barrier discharge plasma [12]. The polymerization rate was found to be dependent not only on the discharge power but also the properties of the surface. Machala *et al.* observed the deposition of solid product from a pilot-scale test on VOC mixtures (mostly cyclohexanone) [13]. They measured the solid products using DRIFT and high-precision liquid chromatography (HPLC) and found amino acids as the main component of the solid products. Nolan and his colleagues directly measured nanometer-sized aerosols in negative point corona discharges [14,15]. Later, Borra *et al.* studied aerosol formation in point-to-plane DC corona for both polarities [16], and various types of discharges (such as streamers, spark, and dielectric barrier discharge) as well [17]. Formation of submicron-sized aerosols and their chemical composition have been confirmed using FTIR and SEM in the X-ray irradiation of benzene or acetylene (below about 1,000 ppm) in air [18]. Despite big advances in the understanding of nonthermal plasma chemistry over the past two decades, the mechanism for the aerosol formation remains elusive. A practical viewpoint, *i.e.*, the consideration of aerosols as unwanted byproducts, has also hindered studies focusing on the fundamental processes of gas-to-particle conversion.

This paper presents experimental results on the aerosol formation from benzene in nonthermal plasma-induced chemical reactions. The size distribution and number concentration were evaluated using a differential mobility analyzer (DMA) and a Faraday cup (FC). The morphology and the chemical composition of the aerosol were measured with scanning electron microscopy (SEM) and a diffuse reflectance infrared Fourier transform (DRIFT) spectrometer, respectively. The similarity of NTP with atmospheric chemistry with regards to aerosol formation and the application of NTP as a tool for the accelerated testing of VOC-to-particle conversion will also be discussed.

## 2. Experimental

**Figure 1.**
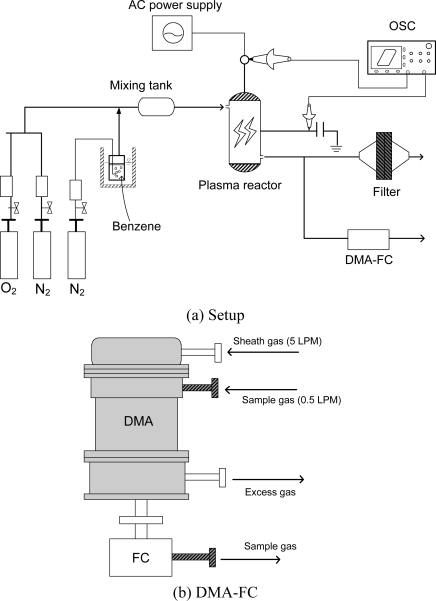
Schematic diagram of the experimental setup **(a)** and differential mobility analyzer (DMA) and Faraday cup (FC) for aerosol measurement **(b)**.

Figure 1 shows the experimental setup (a) and the DMA (differential mobility analyzer; Wyckoff Co., Ltd.) equipped with a Faraday cup (FC) for the aerosol measurement. Since the size range was found to below 100 nm in the previous study [19], the DMA was optimized to measure aerosols smaller than 100 nm. The experimental setup consisted of the plasma reactor, power supply, oscilloscope, and gas cylinders. A cylindrical surface discharge plasma reactor was used in this study. The inner diameter and effective length of the quartz tube were 15 mm and 200 mm, respectively. A coil-type electrode (0.45 mm diameter) was set at the inner surface of the quartz tube, which served as a high-voltage electrode. Silver paste was painted on the outer surface of the reactor as a ground electrode. The plasma reactor was energized with AC high-voltage. The input signal from the function generator (Tektronix, AFG 310) was amplified 2000-fold by a Trek 20/20B amplifier, and then applied to the plasma reactor. The charge Q was measured with a capacitor of 1μF connected in series to the grounded line of the plasma reactors. The discharge power (W) dissipated in the plasma reactor was measured by V-Q Lissajous figure method. Specific input energy (SIE), discharge power (W) transferred to the unit gas flow rate (liters per min; LPM), is calculated from the following equation:

(1) $$\text{Specific}\;\text{input}\;\text{energy}\;(J/L)=\frac{\text{discharge}\;\text{power}\;(\text{watt})}{\text{gas}\;\text{flow}\;\text{rate}\;(L/\text{min})}\times 60$$

The units of J/L can be converted into Wh/Nm^3^ by multiplying by a factor of 3.6. Applied voltage and discharge current were measured using a digital oscilloscope (Tektronix, TDS 3032) connected with a high voltage probe (Tektronix, P6015A) and an current probe (Pearson Electronics Inc., Model 2877), respectively.

The shape of the aerosol was measured using field-emission scanning electron microscopy (FE-SEM; TOPCON Co. Model DS-720). The discharge with N_2_ gas became unstable with time as the deposition of solid products occurred on the inner surface of the plasma reactor. Considering this property, aerosol sampling time was varied from about 30 min for N_2_ to about 120 min for air. The chemical structure of the aerosol was measured using a Diffuse Reflectance Infrared Fourier Transform (DRIFT) spectrometer (Perkin Elmer, Spectrum One). The diffractive reflectance cell was purged with pure nitrogen (2 LPM) during the measurements. The spectrum data were taken by averaging 60 scans with the resolution of 2 cm^−1^. Background spectrum was measured using a clean filter instead of normal method using KBr. A DMA (differential mobility analyzer; Wyckoff Co., Ltd.) equipped with a Faraday cup (FC) was used to measure the size distribution and the number concentration of aerosols. The DMA used in this study can measure aerosol size from 1 nm to 90 nm, depending on the flow rate and applied voltage. To reduce aerosol loss during the transportation, Tygon tube or stainless steel was used as tubing. The current in the FC was measured with a femto-ampere electrometer. The sample gas to the DMA was injected at 0.5 LPM while the sheath gas was at 5 LPM. The distance between the outlet of the plasma reactor and the inlet of the DMA was about 20 cm.

All experiments have been done at atmospheric pressure and room temperature. Gas flow rate was set at 2 LPM under standard condition (273 K, 0.1 MPa) unless otherwise noted. This corresponds to a gas residence time of 1.06 s. Synthetic air or nitrogen were adjusted using mass flow controllers and gas cylinders, which also ensure conditions free from background aerosols. The purities of nitrogen and oxygen were 99.999% and 99.9%, respectively. A bottle containing deionized water was immersed in a water bath maintained at 25 °C to sustain a constant water content of 0.5%. Benzene concentration was adjusted by the same bubbling method and the resulting benzene-laden N_2_ gas was mixed with the main O_2_/N_2_ gas stream. The water vapor content was measured with a dew point hygrometer (General Eastern, Hygro-M4). Benzene concentration was measured with on-line FTIR equipped with a long-path gas cell (6.4 m). A PTFE membrane filter (0.1 μm; ADVANTEC Inc.) was used in sampling aerosols for the DRIFT spectrometer. Qualitative filter paper (No. 2, ADVANTEC Inc.) was used in sampling aerosols for the FE-SEM measurement.

## 3. Results and Discussion

### 3.1. Chemical Conversion of Benzene

Gas-phase benzene was fed into the plasma reactor and its conversion rate and byproducts were measured using the on-line FTIR spectrometer. Figure 2 shows the conversion of benzene (a) and the carbon balance as a function of SIE (b). Gas-phase products determined from the FTIR spectrometer were CO_2_, CO and HCOOH (formic acid). Based on the quantitative FTIR measurement, carbon balance was calculated from the following equation:

(2) $$\text{Carbon balance}\;(\%)=\frac{\left[\text{CO}\right]+\left[{\text{CO}}_{2}\right]+\left[\text{HCOOH}\right]}{6({\left[C_{6}H_{6}\right]}_{0}-\left[C_{6}H_{6}]\right)}\times 100$$

where [C_6_H_6_]_0_ and [C_6_H_6_] indicate the inlet and the outlet concentrations of benzene, respectively. The concentration of benzene showed an exponential decay with SIE. The removal efficiency reached 50% at about 360 J/L. On the other hand, the carbon balance was about 60% at SIE below 100 J/L, and monotonically increased with further increases of SIE.

**Figure 3.**
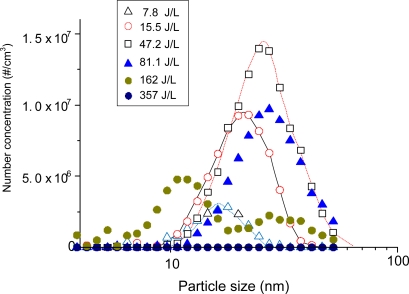
Size distribution of aerosol according to specific input energy to the plasma reactor. (204 ppm benzene in humid air).

The missing parts in carbon balance are often found as solid products (including aerosols) on the reactor wall or on the tubing. Figure 3 shows the size distribution of the aerosol according to the energy input to the plasma reactor. It should be noted that aerosol formation did not occur in the absence of benzene in this study. The formation of solid products in benzene-N_2_ mixtures was so rapid that the stable operation of the reaction was hampered with time. For this reason, aerosol measurements with DMA-FC were only done for air mixtures. Aerosol formation was detected even at low SIE of 7.8 J/L. The peak size and number concentration kept increasing up to 47.2 J/L, and reached 25 nm and 1.4 × 10^7^ particles/cm^3^, respectively. When the SIE was increased to 81.1 J/L the number concentration decreased by about 30% without changing the size distribution. These growth and decay characteristics of aerosols with SIE can be explained by the branching of chemical reaction. At low SIE range below about 50 J/L, formation and growth of aerosol are occurring dominantly. On the other hand, the produced aerosols may further undergo oxidation that dominates at SIE values higher than about 50 J/L. The unimodal distribution changed to a bimodal distribution at 162 J/L. Aerosols completely disappeared at 357 J/L, which is consistent with the carbon balance data in Figure 2(b). This observation also indicates that the aerosols are composed mostly of organic compounds.

**Figure 4.**
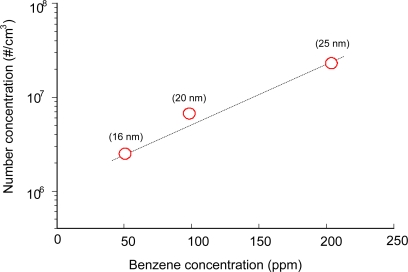
Influence of benzene concentration on the number concentration of aerosols (humid air). The figures in parentheses indicate the peak size.

Figure 4 shows the influence of benzene concentration on the number concentration of the aerosol. The SIE was fixed at around 50 J/L and the size distribution and the number concentration were measured with the DMA-FC. As expected, number concentration of aerosol was largely influenced by the benzene concentration. When the benzene concentration was increased from 50 ppm to 200 ppm, the number concentration increased by a factor of 10. At the same time, peak sizes were also increased with the inlet concentration of benzene. In our previous works using a scanning mobility particle sizer (SMPS, TSI), surface discharge produced aerosols with a lower number concentration of 5.9 × 10^4^ particles/cm^3^ with a larger peak size of 39 nm [19]. Besides the different measuring instrument, the long distance (3 m) before entering the SMPS is believed to be the main reason for the difference in size distribution. Coagulation is a typical characteristic of aerosols, especially in the nanometer-sized range, resulting in the growth in size. The short distance (20 cm) between the plasma reactor and the DMA provides information about aerosols much closer to those in the plasma reactor. Since the gas mixtures fed to the plasma reactor were free from background aerosols or NH_3_, ions generated in the plasma reactor are expected to play an important role in this process. Ion-induced nucleation is well-known to play an important role in the gas-to-particle process in a plasma environment [20]. If we assume that the main chemical reactions occur during the discharge period, the density of charge carrier (N_e_) during the reactions can be calculated from the measured discharge current density, J (A/cm^2^) [21,22]:

(3) $$J=eN_{e}v$$

where *e* and *v* are the elementary charge (1.6 × 10^−19^ C) and drift velocity, respectively. For the typical conditions in this study (E = 10 kV/cm, average discharge current = 5 mA, reduced electric field (E/N) = 80 Td) the N_e_ in the plasma reactor was calculated to about ∼10^8^/cm^3^. Since the diameter of microdischarge (streamer) is about 100 μm, the local ion density in the reaction zone is expected to reach up to ∼10^12^/cm^3^.

### 3.2. Aerosol Analysis

**Figure 5.**
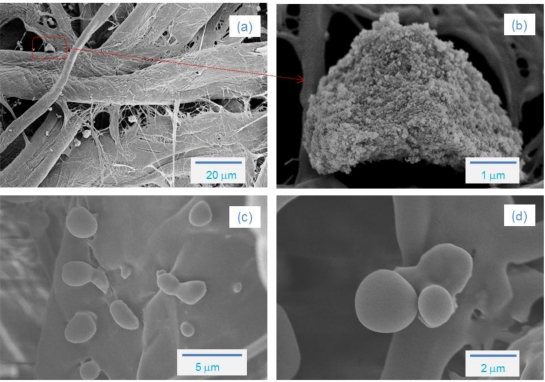
FE-SEM photos of the aerosol; **(a)** and **(b)** humid air, **(c)** and **(d)** humid N_2_. Benzene concentration was about 250 ppmv.

Figure 5 shows FE-SEM photos of the aerosol collected downstream of the plasma reactor. In the case of air mixtures, the size of aerosols (a) collected on the filter was in the 0.5–2.0 μm range. The FE-SEM photo with further magnification, (b) ×20,000, clearly indicated that the each particle is a collection of nanometer-sized aerosols, which is consistent with the DMA measurement. Under nitrogen conditions, deposition of solid products with dark-brown colors can be observed near the outlet of plasma reactor, even with the naked eye. The FE-SEM photos indicated that the aerosols formed in a N_2_ environment had smooth surfaces and irregular size (1–5 μm). In an early review by Fomin it was indicated that the reaction of benzene with active nitrogen produced nitrogen-containing polymers [23].

**Figure 6.**
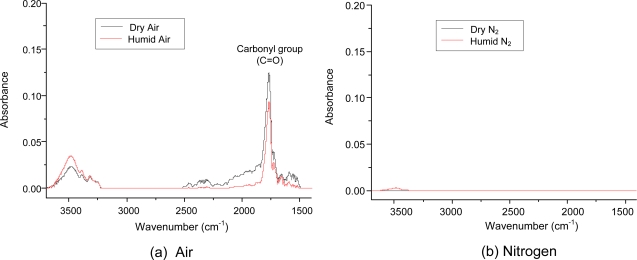
Chemical analysis of aerosol using DRIFT spectrometer; **(a)** in air **(b)** in N_2_. Benzene concentration was about 250 ppmv.

The chemical composition of the aerosol was measured with the DRIFT spectrometer, and the data are shown in Figure 6. To avoid water adsorption on the filter, a PTFE membrane filter used in aerosol sampling for the DRIFT measurements. Under air conditions, water vapor did not influence the DRIFT spectrum. The most prominent absorption band was at 1,650–1,800 cm^−1^, which was assigned to a carbonyl group (C=O) [24]. It should be noted that the carbonyl group peak did not appear under nitrogen conditions. This observation supports that the oxygen plays more dominant role than the water vapor in the formation of C=O groups. The large absorption of the carbonyl group also provided firm evidence that the ring cleavage products dominated in the aerosol. The broad spectral band of 3,200–3,700 cm^−1^ was assigned to water molecules on the surface (stretching vibrations of hydroxyl groups) [25]. This peak was not observed in dry nitrogen. The C=C bond in the aromatic ring (1,550 cm^−1^) was not observed under any tested conditions. This result indicates that the ring-cleavage products are the major compounds of the aerosols.

Electron-beam (e-beam) irradiation of gas mixtures containing VOCs also produces nanometer-sized aerosols [26,27]. Hakoda *et al.* measured the components of a 50-keV e-beam induced aerosol from *o*-xylene using atmospheric pressure ionization mass spectrometry (API-MS) [28]. The main component in the aerosol were found to be alkyl acids and aldehydes, which are ring-cleavage products. Interestingly, the API-MS signals of *m/z* 139–203 exhibited a constant interval of *m/z* 16, which corresponds to atomic oxygen. This result is consistent with the DRIFT spectrum in this study. The formation of carbonyl group and the undetectable levels of aromatic rings suggested that the oxygen species are involved in the ring-cleavage process. Interestingly, the results obtained with NTP or e-beam are quite consistent with those reported in atmospheric chemistry. For example, Forstner *et al.* studied the formation of SOA from seven aromatic hydrocarbons in 60 m^3^ outdoor smog chamber experiments and reported the formation unsaturated anhydrides (2,5-furandione, 3-methyl-2,5-furandione, 3-ethyl-2,5-furandione) as the predominant compounds of aerosols [29]. They explained the results using the gas-phase mechanisms involving ring fragmentation. Ring fragmentation reactions were also found to be important in the UV photooxidation of toluene and *o*-xylene [30,31]. Another chamber experiment (9.0 and 11.3 m^3^) for the mixture of aromatic VOCs also reported the significant formation of carbonyl groups in SOA [25].

Although differing in concentration, NTP and atmospheric chemistry are based on similar chemical reactions involving CAS such as OH radicals, HO_2_ radicals, and O_3_. Plasma can easily control the concentration of CAS. In this sense, NTP technology can be also used as a tool for the accelerated flow-through testing of gas-to-particle conversion of various VOCs. Preliminary results of this study provided some supporting evidence that the control of aerosol growth is possible by adjusting the energy input to the reactor.

## 4. Conclusions

Aerosol formation from benzene in a surface discharge nonthermal plasma reactor was investigated using DMA-FC, FE-SEM, and a DRIFT spectrometer. The influences of basic parameters such as specific input energy, inlet concentration, gas composition have been measured and discussed. Since there is a close similarity between atmospheric chemistry and nonthermal plasma chemistry in principle, further information along these lines would be helpful for a better understanding of SOA formation in the atmosphere.

## Figures and Tables

**Figrue 2.**
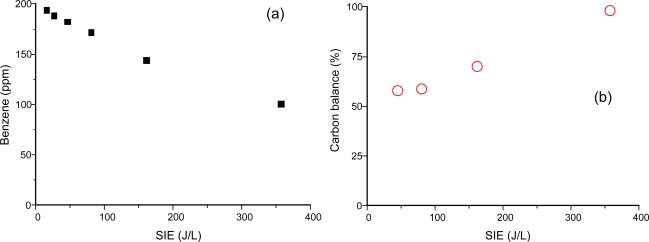
Benzene removal in the surface discharge plasma reactor; **(a)** removal efficiency, **(b)** carbon balance.

**Table 1.** Typical parameters in atmospheric chemistry and plasma chemistry (in air).

**Parameters**	**Atmospheric Chemistry**	**Plasma Chemistry**
Temperature	273 ∼ 293 K	<373 K
OH radicals	∼10^6^ cm^−3^	∼10^15^ cm^−3^
O_3_	∼10^−1^ ppm	∼10^3^ ppm
UV intensity	∼10^2^ mWcm^−2^	∼μWcm^−2^
NOx	<ppm	∼10^2^ ppm
Reactant	∼ppb	∼ppm
